# Detecting the Land-Cover Changes Induced by Large-Physical Disturbances Using Landscape Metrics, Spatial Sampling, Simulation and Spatial Analysis

**DOI:** 10.3390/s90906670

**Published:** 2009-08-26

**Authors:** Hone-Jay Chu, Yu-Pin Lin, Yu-Long Huang, Yung-Chieh Wang

**Affiliations:** Department of Bioenvironmental Systems Engineering, National Taiwan University, 1, Sec. 4, Roosevelt Rd., Da-an District, Taipei City 106, Taiwan; E-Mails: honejaychu@gmail.com (H.-J.C.); morris0109@hotmail.com (Y.-L.H.); b92602015@ntu.edu.tw (Y.-C.W.)

**Keywords:** spatial analysis, Latin hypercube sampling, conditional simulation, landscape metrics, land cover change, remotely sensed images, geostatistics, Google Earth

## Abstract

The objectives of the study are to integrate the conditional Latin Hypercube Sampling (cLHS), sequential Gaussian simulation (SGS) and spatial analysis in remotely sensed images, to monitor the effects of large chronological disturbances on spatial characteristics of landscape changes including spatial heterogeneity and variability. The multiple NDVI images demonstrate that spatial patterns of disturbed landscapes were successfully delineated by spatial analysis such as variogram, Moran’I and landscape metrics in the study area. The hybrid method delineates the spatial patterns and spatial variability of landscapes caused by these large disturbances. The cLHS approach is applied to select samples from Normalized Difference Vegetation Index (NDVI) images from SPOT HRV images in the Chenyulan watershed of Taiwan, and then SGS with sufficient samples is used to generate maps of NDVI images. In final, the NDVI simulated maps are verified using indexes such as the correlation coefficient and mean absolute error (MAE). Therefore, the statistics and spatial structures of multiple NDVI images present a very robust behavior, which advocates the use of the index for the quantification of the landscape spatial patterns and land cover change. In addition, the results transferred by Open Geospatial techniques can be accessed from web-based and end-user applications of the watershed management.

## 1. Introduction

The interest in land cover analysis at regional to global scales has grown dramatically in the last decade [1]. To ensure a sustainable management of natural resources, it is necessary to understand and quantify the landscape change processes. Patterns of landscape change are the results of complex interactions between physical, biological and social forces [2]. To understand and predict change processes, one needs to monitor and characterize spatial patterns of land use/land cover change [3]. Therefore, the methods for proving the sufficient information to understand the land cover change, such as sampling and simulation are very important. Remote sensing has emerged as the most useful data source for quantitatively measuring land-cover changes at the landscape scale. The dynamics of change processes can be investigated through temporal series of remote sensing data and by analyzing change trajectories [3,4]. Remotely sensed data can describe surface processes, including landscape dynamics and land cover change, as such data provide frequent spatial estimates of key environmental variables [5]. Land cover change is regarded as the single most important variable of global change affecting ecological systems with an impact on the environment [6]. Satellite image data have become an important source of information for monitoring the vegetation conditions, land use and land cover change [7]. The most widely used vegetation index in this context is NDVI, the normalized difference vegetation index, which is a function of red and near-infrared spectral bands [8]. The NDVI value indicates a level of photosynthetic activity [9]. The change areas can be identified through the subtraction of the NDVI image of one date from the NDVI image of another date [10]. Significant differences of NDVI images in landscape changes, such as landslides are induced by a disturbance. In addition, the landscapes are a composite of complex nature and biophysical forces that are manifested on the landscape through the composition and spatial organization of land cover and variations in NDVI. Multi-temporal NDVI images are practical for monitoring vegetation dynamics on a regional scale.

The land cover change areas usually cover only a small portion of the entire image and may not be detected or represented by simple random sampling or systematic sampling unless the sampling intensity is very high. The principle of sampling for land cover analysis is well established [1,10,11]. Random sampling is statistically appealing because of the applicability of the classical statistical procedures. But it is not generally an efficient approach. For this reason, other sampling designs such as stratified random, systematic grid, and others may be preferred for land cover analysis. However, the sampling problem is complicated by the nature of fine resolution satellite data. For reasons of costs and efficiency of using the acquired data, it is much preferable to select scenes that will make the greatest contribution to the characterization of land cover over the entire domain [1]. Thus, Latin hypercube sampling (LHS) was used to obtain reference data for this study. LHS was initially developed for the purpose of Monte-Carlo simulation, efficiently selecting input variables for the models [12,13]. Conditional Latin hypercube sampling (cLHS), which is based on the empirical distribution of original data, provides full coverage of each variable by maximally stratifying the marginal distribution and ensuring a good spread of sampling points [14]. The cLHS is an effective way to replicate the distribution of variables compared with random sampling and stratified spatial sampling, and ensures the correlation of sampled variables to replicate the original data [14,15]. After cLHS sampling, geostatistical conditional simulations with the sampling data can be applied to simulate the spatial variability and spatial distribution of interest [16]. For sampled data, a geostatistical conditional simulation technique, such as sequential Gaussian simulation (SGS), can be applied to generate multiple realizations, including an error component, which is absent from classical interpolation approaches [17–20]. Sequential simulation procedures generate simulated values using a Monte Carlo approach at each node of the simulation grid visited sequentially according to a random path. SGS realization varies greatly in space on the condition of original data and all previously simulated values [19,21].Moreover, development of efficient procedures for designing information-effective samplings and simulations is an essential task for more accurately understanding the spatial distribution.

Spatial analysis (i.e. Moran’I, and variogram) delineates spatial variations and patterns of land cover from remote sensing images [6,22,23]. Variograms are crucial to geostatistics. Both are functions related to the variance of spatial separation and provide a concise description of the scale and pattern of spatial variability [24]. Samples of remotely sensed data (e.g., satellite or air-borne sensor imagery) can be employed to construct variograms for remotely sensed research [24]. Moreover, Moran’I and variograms have been used widely to understand the nature and causes of spatial variation within an image [22,25,26]. The Moran’I and variogram characterize and quantify spatial autocorrelation, heterogeneous spatial components (spatial variability and spatial structure) of a landscape and the spatial heterogeneity of vegetation cover at the landscape level [23,25,26]. Furthermore, landscape metrics have been used increasingly to assess land-use change in the last decade [6,27–30]. Variables that characterize landscape patterns, such as the number, area, and spatial patterns of different patch types, change when the land use/ land cover change is altered. Landscape composition, configuration, and connectivity are primary descriptors of landscape patterns [27], and can be quantified using spatial landscape indices or metrics. Landscape composition refers to the characteristics associated with the variety and abundance of patch types within a given landscape. Spatial configuration of a landscape denotes the spatial characteristics and arrangement, position, or orientation of patches within a class or landscape [31]. In addition, landscape metrics have proved effective in land-use planning and design because they can characterize the landscape pattern in the design alternatives [6,32,33]. Accordingly, monitoring, delineating and sampling landscape changes, spatial structure and spatial variation induced by large physical disturbances are essential to landscape management and restoration, and disaster management in Taiwan. Earthquakes have long been recognized as a major cause of landslides [34,35]. The earthquake seriously damaged the vegetation and landslides in the region [35–37]. Additionally, typhoons are the main external triggering factor to debris flow and are extremely important natural disturbances that characterize the structure, function and dynamics of many ecosystems [38–40].Typhoons that bring tremendous amounts of rainfall hit Taiwan every year from July to October [41]. During 1996–2004, large disturbances in the following sequence impacted central Taiwan: such as typhoon Herb (August 1996); the Chi-Chi earthquake (September 1999); typhoon Xangsane (November 2000); typhoon Toraji (July 2001); typhoon Dujuan (September, 2003), and typhoon Mindulle (June 2004) [41]. After the Chi-Chi earthquake, the expansion rate of landslide areas increased 20-fold in central Taiwan [42].The influences of large physical disturbances on ecosystem structure and function have garnered considerable attention [43–46]. Numerous extension cracks, which accelerate landslides during downpours, were generated on hill slopes during the Chi-Chi earthquake [37,40,47]. The landslides are the natural hazards and the resulting in a variety of human and eco-environmental impacts [20].

In the study, the NDVI data derived from SPOT images before and after the Chi-Chi earthquake in the Chenyulan basin of Taiwan, as well as images before and after four large typhoons (Xangsane, Toraji, Dujuan and Mindulle) were analyzed to identify the landscapes change caused by these major disturbances. For the representation of the changes of Multi-temporal NDVI data, we used the hybrid methods including spatial sampling, simulation, spatial analysis and landscape metrics in the space-time data analysis. The spatial analyses identify the landscapes change and delineate spatial variations of NDVI images before and after large physical disturbances in central Taiwan. Moreover, conditional LHS (cLHS) schemes with NDVI images were used to select spatial samples from actual NDVI images to detect landscape changes induced by a series of large disturbances. Furthermore, the best cLHS samples selected with the NDVI values were used to simulate NDVI distributions using SGS. Finally, the analyses are used to evaluate the approaches for defining a sufficient sampling size to capture the spatial variability of the land cover. The study can be used to understand the NDVI spatial structures within an image domain for the watershed management.

## 2. Methods and Materials

### 2.1. Study Area and Remote Sensing Data

**Figure 1.**
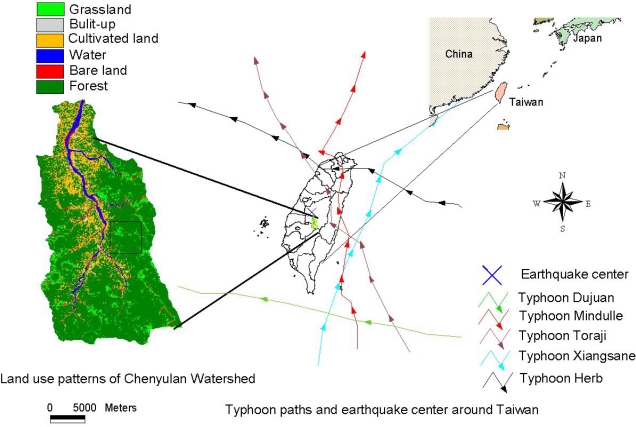
Typhoon paths and Chi-Chi earthquake center are around Taiwan, and land use patterns of the study area.

The Chenyulan watershed, located in central Taiwan, is a classical intermountain watershed which is traversed by the Chenyulan stream in the south to north direction. The average altitude and area of this watershed are 1,540 m and 449 km^2^, respectively (Figure 1). Differences in uplifting along the fault generated abundant fractures over the watershed and resulted in an average slope of 62.5% and relief of 585 m/km^2^. Moreover, the main course of the Chenyulan stream had a gradient of 6.1%, and more than 60% of its tributaries had gradients exceeding 20%. In this area, slates and meta-sandstones are the dominant lithologies in the metamorphic terrains [25,42]. Based on the relative amounts of slate and meta-sandstone, the metamorphic strata in the eastern part of the study area are divided into four parts: Shihpachuangchi, Tachien Meta-Sandstone, Paileng Meta-Sandstone, and Shuichangliu [25,42]. Figure 1 also shows the six land cover categories in the area on November 19, 2004 which include forest, grassland, cultivated land, water, bare land, and built-up. The details could be referred to [48]. The study area with dimensions of 5 × 5 km^2^ (250 × 250 pixels) was selected from the upstream of the large debris flood announced in the watershed, as shown in Figure 1.

In this paper, the NDVI changes in land cover reflect the natural consequences of typhoons and earthquakes. At 01:47′12.6″ on September 21, 1999, an earthquake of magnitude 7.3 on the Richter scale and with a focal depth of 8.0 km struck central Taiwan. The cause of this event, known as the Chi-Chi earthquake, was movement of the CheLungPu fault. The epicenter was located at 23.87°N and 120.75°E, near the Chenyulan watershed in south Nantou County. The magnitude of the earthquake was estimated to be ML = 7.3 (ML: Local Magnitude or Richter Magnitude), and the rupture zone, defined by the aftershocks, measured about 80 km north-south by 25–30 km downdip [47,49]. Iso-contour maps of the earthquake’s magnitude were reproduced from the Central Weather Bureau (Figure 1) [50]. After the earthquake, the center of typhoon Xiangsane moved from south to north through eastern Taiwan from October 31, 2000 to November 1, 2000 [51], with a maximum wind speed of 138.9 km/hr and a radius of 250 km (Figure 1). The maximum daily rainfall was 550 mm/day. The Toraji typhoon swept across central Taiwan from east to west on July 30, 2001 [52] (Figure 1). The typhoon brought extremely heavy rainfall, from 230 to 650 mm/ day, and triggered numerous landslides in Taiwan. Typhoon Toraji became a tropical storm after crossing Taiwan; however it brought 339 to 757 mm of total accumulated rainfall in the watershed [52] (Figure 1). From August 31, 2003 to September 2, 2003, typhoon Dujuan was unusual in the double eye with a maximum wind speed of 165.0 km/hr, a radius of 200 km and maximum rainfall 200 mm/hr. From June 29, 2004 to July 2, 2004, Mindulle with maximum wind speed of 200.0 km/hr, a radius of 200 km. Severe rainfall (with a maximum amount of 787 mm) occurred over central-southwestern Taiwan (Figure 1) [53].

### 2.2. NDVI

The NDVI is an empirically derived index used to estimate plant biomass through the integration of the red–visible and near-infrared spectral regions to represent plant pigmentation and chlorophyll content, respectively, in the characterization of land cover conditions [28]. Seven cloud-free SPOT images (1996/11/08, 1999/03/06, 1999/10/31, 2000/11/27, 2001/11/20, 2003/12/17 and 2004/11/19) of the Chenyulan watershed were purchased from the Space and Remote-sensing Research Center, Taiwan. The NDVI images of the study area were generated from SPOT HRV images with a resolution of 20 m according to the following equation:

(1) $$\mathrm{NDVI}=\frac{\mathrm{NIR}-R}{\mathrm{NIR}+R}$$

where NIR and R are near-infrared and visible-red spectral data, respectively. The NDVI values range from −1 to +1; a high NDVI value represents a large amount of high photosynthesizing vegetation [53].

### 2.3. Landscape Matrices

A wide variety of indices developed to characterize landscapes has often been applied to study spatial landscape patterns in landscape ecology; they can be categorized into: area/density/edge, shape, core area, isolation/proximity, contrast, contagion, connectivity, and diversity metrics [31]. Since landscape metrics can be used to analyze the spatial and temporal changes in landscape patterns, they provide a quantitative approach for studying the land cover change through the measurement of spatial and temporal variations in these metrics. To assess changes in land-use patterns, the following landscape metrics were calculated using the Patch Analyst in the GIS software ArcView 3.2a [55] is designed to compute a wide variety of landscape metrics for categorical map patterns. In this study, the following metrics were used (Table 1): the number of patches (NP), mean patch size (MPS), Patch Size Standard Deviation (PSSD), Patch Size Coefficient of Variance (PScov), Edge Density (ED), mean shape index (MSI) and mean nearest neighbor (MNN) [31].

### 2.4. Variogram

In geostatistical methods, variograms can be used to quantify the observed relationship between the values of samples and the proximity of samples [19]. Following the work of Garrigues *et al*. [23], Garrigues *et al*. [26] and Lin *et al*. [35], NDVI data are considered values of punctual regionalized variable. An experimental variogram for interval lag distance class *h*, *γ*(*h*), is represented by:

(2) $$\gamma \;(h)=\frac{1}{2n(h)}\sum_{i=1}^{n(h)}{\left[Z\left(x_{i}+h\right)-Z\left(x_{i}\right)\right]}^{2}$$

where *h* is the lag distance that separates pairs of points; *Z*(*x*) is NDVI value at location x, and Z(*x* + *h*) is NDVI value at location *x* + *h; n*(*h*) is the number of pairs separated by lag distance *h*.

### 2.5. Conditional Latin hypercube

The cLHS procedure represents an optimization problem: given N sites with ancillary data (Z), select n sample sites (*n* << *N*) such that the sampled sites form a Latin hypercube. For k continuous variables, each component of Z (size, *N* × *k*) is divided into n (sample size) equally probable strata based on their distributions, and z (size *n* × *k*) is a sub-sample of Z. The procedures of the cLHS algorithm [14] are as follows:

1. Divide the quantile distribution of Z into n strata, and calculate the quantile distribution for each variable, $q_{j}^{i},...,q_{j}^{n+1}$. Calculate the correlation matrix of Z (C).
2. Pick n random samples from N; calculate the correlation matrix of z (T).
3. Calculate the objective function. The overall objective function combines to three components of the objective function (*O*_1_, *O*_2_, and *O*_3_). For general applications, all weightings are set to equal for all components of the objective function.
  - For continuous variables,
    (3) $$O_{1}=\sum_{i=1}^{n}\sum_{j=1}^{k}\left|\eta \left(q_{j}^{i}\leq z_{j}\leq q_{j}^{i+1}\right)-1\right|$$
    where $\eta \left(q_{j}^{i}\leq z_{j}\leq q_{j}^{i+1}\right)$ is the number of *z_j_* that falls between quantiles $q_{j}^{i}$ and $q_{j}^{i+1}$
  - For categorical data, the objective function is to match the probability distribution for each class of
    (4) $$O_{2}=\sum_{j=1}^{c}\left|\frac{\eta \prime \left(z_{j}\right)}{n}-k_{j}\right|$$
    where *η’*(*z_j_*) is the number of z that belongs to class j in sampled data, and *k_j_* is the proportion of class j in Z.
  - To ensure that the correlation of the sampled variables will replicate the original data, another objective function is added:
    (5) $$O_{3}=\sum_{i=1}^{k}\sum_{j=1}^{k}\left|c_{\mathrm{ij}}-t_{\mathrm{ij}}\right|$$
    where c is the element of C, the correlation matrix of Z, and t is the equivalent element of T, the correlation matrix of z.
4. Perform an annealing schedule:M = exp [–Δ*O*/T], where Δ*O* is the change in the objective function, and T is a cooling temperature (between 0 and 1), which is decreased by a factor d during each iteration.
5. Generate a uniform random number between 0 and 1. If *rand* < *M*, accept the new values; otherwise, discard changes.
6. Try to perform changes: Generate a uniform random number rand. If *rand* < *P*, pick a sample randomly from z and swap it with a random site from unsampled sites r. Otherwise, remove the sample(s) from z that has the largest $\eta \left(q_{j}^{i}\leq z_{j}\leq q_{j}^{i+1}\right)$ and replace it with a random site(s) from unsampled sites r. End when the value of P is between 0 and 1, indicating that the probability of the search is a random search or systematically replacing the samples that have the worst fit with the strata.
7. Go to step 3. Repeat steps 3–7 until the objective function value falls beyond a given stop criterion or a specified number of iterations.

### 2.6. Sequential Gaussian Simulation

The SGS assumes a Gaussian random field, such that the mean value and covariance completely characterize the conditional cumulative density function [56]. During the SGS process, Gaussian transformation of available measurements is simulated, such that each simulated value is conditional on original data and all previously simulated values [21,57]. A value simulated at a one location is randomly selected from the normal distribution function defined by the kriging mean and variance based on neighborhood values. Finally, simulated normal values are back-transformed into simulated values to yield the original variable. The simulated value at the new randomly visited point value depends on both original data and previously simulated values. This process is repeated until all points have been simulated. In sequential simulation algorithm, modeling of the N-point cumulative density function (ccdf) is a sequence of N univariate ccdfs at each node (grid cell) along a random path [58]. The sequential simulation algorithm has the following steps [58]:

1. Establish a random path that is visited once and only once, all nodes {*x_i_*, *i* = 1, Λ, N} discretizing the domain of interest Doman. A random visiting sequence ensures that no spatial continuity artifact is introduced into the simulation by a specific path visiting N nodes.
2. At the first visited N nodes *x*_1_:
  - Model, using either a parametric or nonparametric approach, the local ccdf of *Z*(*x*_1_) conditional on n original data {*Z* (*x_α_*), *α* = 1,Λ, *n*} *F_Z_* (*x*_1_; *z*_1_|(*n*)) = *prob* {*Z* (*x*_1_) ≤ *z*_1_|(*n*)}
  - Generate, via the Monte Carlo drawing relation, a simulated value *z*^(*l*)^(*x*_1_) from this ccdf *F_Z_* (*x*_1_: *z*_1_|(*n*)), and add it to the conditioning data set, now of dimension *n* + 1, to be used for all subsequent local ccdf determinations.
3. At the i_th_ node *x_i_* along the random path:
  - Model the local ccdf of *Z*(*x_i_*) conditional on n original data and the *i* − 1 near previously simulated values { *z*^(*l*)^(*x_i_*), *j* = 1,Λ, *i* − 1}:
    (6) $$F_{Z}\;\left(x_{1};z_{i}|\left(n+i-1\right)\right)=\mathrm{prob}\{Z\left(x_{i}\right)\leq z_{i}|\left(n+i-1)\right\}$$
  - Generate a simulated value *z*^(*l*)^(*x_i_*) from this ccdf and add it to the conditioning data set, now of dimension *n* + *i*.
4. Repeat step 3 until all N nodes along the random path are visited.

### 2.7. Moran’s I

Spatial autocorrelation is a useful tool for describing the dependency of spatial patterns. First, spatial structures are described by so-called structure functions [25,59].Moran’s I, which ranges between −1 and +1, is a well known spatial autocorrelation method [60]. The index, I, is calculated as follows:

(7) $$I=\frac{(1/W)\sum_{h=1}^{n}\sum_{i=1}^{n}w_{\mathrm{hi}}\left(y_{h}-\bar{y}\right)\left(y_{i}-\bar{y}\right)}{(1/n)\sum_{i=1}^{n}{\left(y_{i}-\bar{y}\right)}^{2}}$$

where y_h_ and y_i_ denote the values of the observed variable at sites h and I, respectively; and w_hi_ denotes the weight of the variable. The weights, w_ij_, are written in an (n × n) weight matrix W, which is the sum of the weights w_hi_ for a given distance class [61]. Moran’s I is high and positive when a value is similar to adjacent values, and low when a value is dissimilar to adjacent values. In this paper, the global Moran’s I value for the NDVI was calculated to compare the spatial relations of the NDVI among various events. As a result, the phenomenon of spatial autocorrelation of NDVI could be tested.

## 3. Results and Discussion

### 3.1. Statistics and Spatial Analysis of NDVI Images

The NDVI is one of the most popular methods for monitoring vegetation conditions. It has been reported that multitemporal NDVI is useful for classifying land cover and the dynamics of vegetation [19,62,63]. However, the typhoons and earthquakes is a major natural disturbance to land cover change in Taiwan. For example, the Chi-Chi earthquake led to landslides, dammed lakes and a high death toll. Like the typhoons, subsequent rainstorms cause divergent destruction of vegetation; this destruction may be influenced by the rainfall distribution and typhoon path. However, due to destruction of vegetation and increased soil exposure in the Chenyulan watershed, the landslide ratio increased with successive rainstorms and strong earthquakes and resulted in the decreased NDVI values [41].

**Figure 2.**
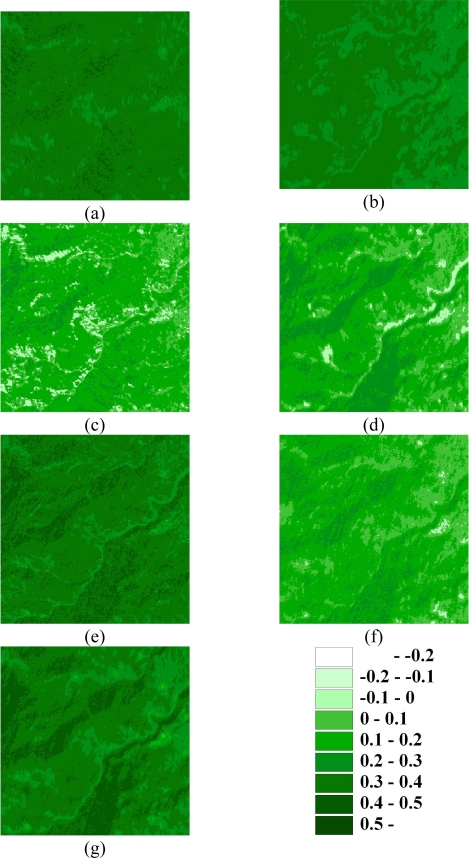
NDVI images of the area on (a) 1996/11/08, (b) 1999/03/06, (c) 1999/10/31, (d) 2000/11/27, (e) 2001/11/20, (f) 2003/12/17, and (g) 2004/11/19.

**Figure 3.**
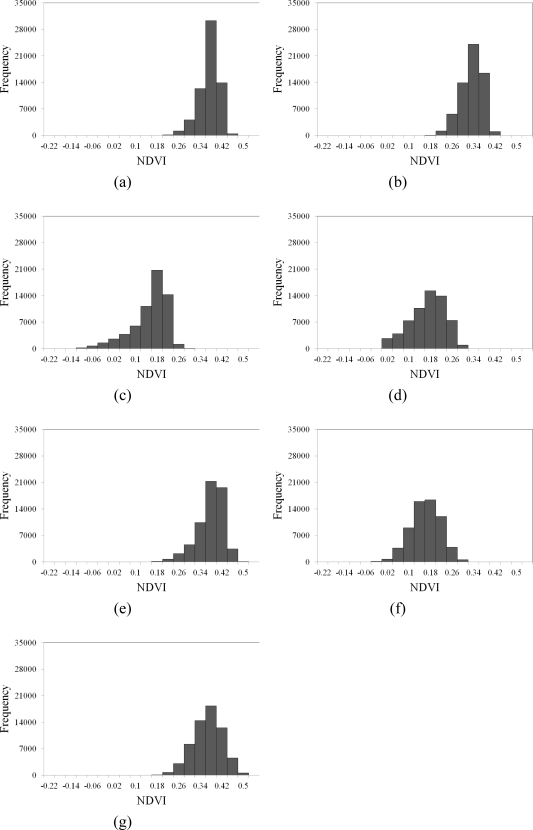
Histogram of original data on (a) 1996/11/08, (b) 1999/03/06, (c) 1999/10/31, (d) 2000/11/27, (e) 2001/11/20, (f) 2003/12/17, and (g) 2004/11/19.

Figure 2 shows the NDVI images of the area on seven events of the large disturbances. Table 2 and Figure 3 summarize the statistics and histograms for seven actual NDVI images of the area before and after disturbances. The lowest mean and minimum NDVI values in 1996–2004 occurred on October 31, 1999, after the Chi-Chi earthquake in the studied area. Moreover, the largest range between minimum and maximum NDVI values also occurred on October 31, 1999, after the Chi-Chi earthquake in the study area. The other negative minimum NDVI values occurred on November 27, 2000, and December 17, 2003, in the study area. The second and third greatest impacts on all landscapes are from typhoons Xangsane (November 2000) and Dujuan (September 2003) in the area, respectively (Figure 2 and Table 2). During the dates, the standard deviations of NDVI values were slightly larger than those on the other dates. These statistical results illustrate that the Chi-Chi earthquake had the largest impact on all landscapes represented by NDVI images for the study area. Unfortunately, typhoon Xangsane that came after the Chi-Chi earthquake was the second disturbance to impact landscape changes in the study area. Numerous extension cracks, which increase the number of landslides during downpours, were generated on hill slopes during the Chi-Chi earthquake [25]. Statistics of remotely sensed images can be used as a basic tool to characterize landscape changes [64–68]. Statistical results illustrate that the effects of disturbances on the watershed landscape in the study area were significantly different in space and time over the entire landscape.

**Figure 4.**
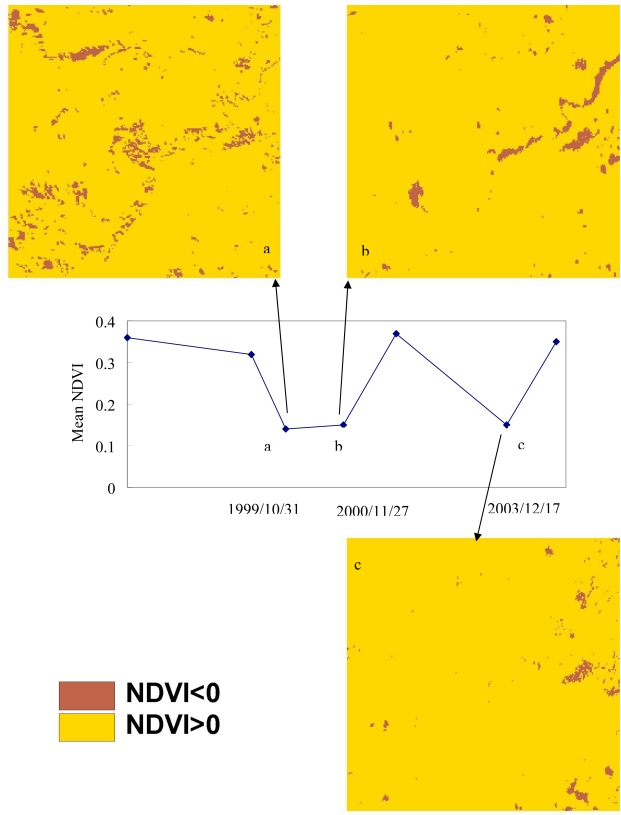
The NDVI classification data in three events.

**Figure 5.**
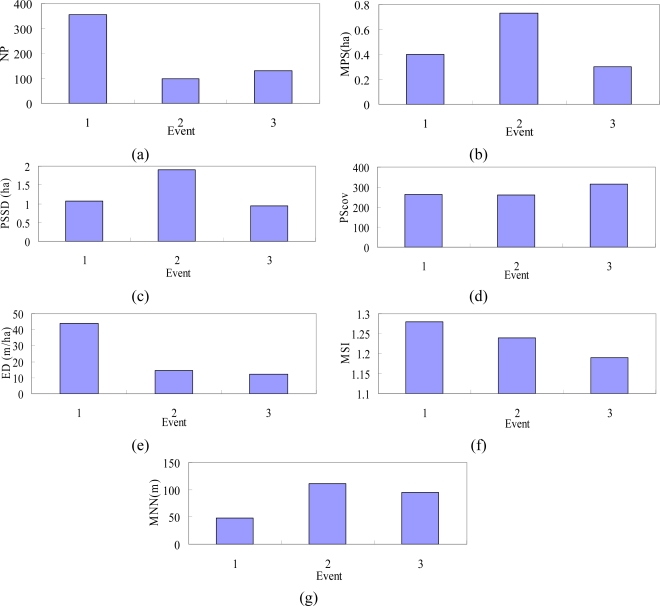
Landscape matrices in (a) the number of patches (NP), (b) mean patch size (MPS), (c) Patch Size Standard Deviation (PSSD), (d) Patch Size Coefficient of Variance (PScov), (e) Edge Density (ED), (f) Mean Shape Index (MSI), (g) Mean Nearest Neighbor (MNN) in the event 1. (1999/10/31), event 2 (2000/11/27) and event 3 (2003/12/17).

The first three lowest mean NDVI images in 1996–2004 occurred on 1999/10/31(Table 2), after the Chi-Chi earthquake, typhoons Xangsane (2000/11/27), and Dujuan (2003/12/17) in the study area. Figure 4 shows mean NDVI varying with time and the classification data in the events for the low mean NDVI values and the brown parts in the images represent sparse vegetation when the NDVI values are less than zero. Landscape metrics were used to assess the damage of the three events include the Chi-Chi earthquake, typhoon Xangsane and Dujuan, and demonstrate the landscape metrics of land-use patterns in the low NDVI class level. The results from mean NDVI calculation revealed a stable plant growth and vegetation recovery tendency of the study area. These results confirm the previous studies that the vegetation keeps on recovering in the landslide areas but earthquake and subsequent rainstorms may impact on the vegetation recovery rates [20,69,70]. Figure 5 shows landscape matrices for (a) the number of patches (NP), (b) mean patch size (MPS), (c) Patch Size Standard Deviation (PSSD), (d) Patch Size Coefficient of Variance (PScov), (e) Edge Density (ED), (f) Mean Shape Index (MSI), (g) Mean Nearest Neighbor (MNN) in the events. Form the analysis of the indexes, the NP and ED for low NDVI class of the earthquake is larger than that of typhoon events. It is discovered that there is a large number of patches and edge density in the earthquake. The value of MPS for low NDVI class is the largest on 2000/11/27, middle on 1999/10/31, and the smallest on 2003/12/17. The shape index (MSI) and edge index (ED) present robust behaviors, which advocate the use of the indexes for the quantification of the overall irregularity of patch shapes and edge in low NDVI class. The MSI for low NDVI class on 2003/12/17 (after typhoon Dujuan) is the smallest and represents that the landslide patch shape is close to the square. Moreover, the MNN for low NDVI on 1999/10/31 (after the Chi-Chi earthquake) is less than that of the typhoon events. Classification data of NDVI used within landscape metrics also have a number of advantages as them give us information on several indices of spatial distribution including patch size, edge, and shape of patches, all of which are theoretically capable of providing different insights on landscape fragmentation after the large chronological disturbances. These indices show the fragmented NDVI patterns due to the natural disturbances in the watershed. Accordingly, the earthquake induces the major and regional damage but the disturbances of NDVI caused by the typhoons are minor and local in the study area.

**Figure 6.**
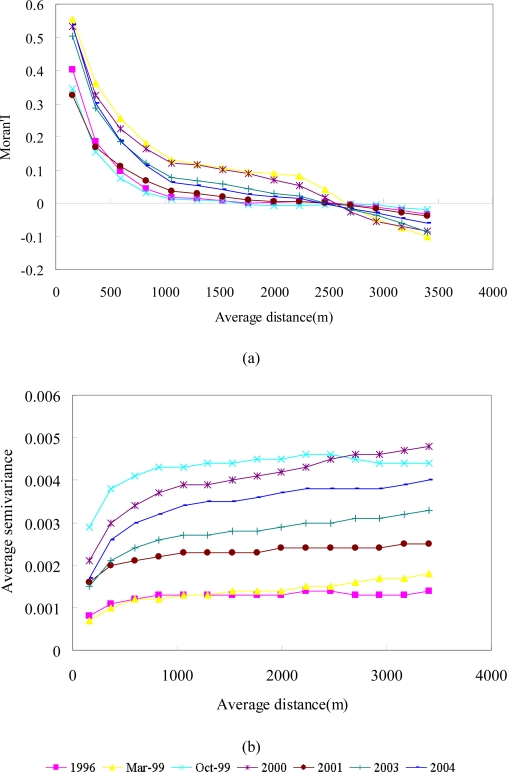
(a) Moran’I and (b) experimental variograms of NDVI images before and after disturbances in the area.

Previous studies that quantified the impact of large disturbances did not evaluate the spatial structures of NDVI images in the study areas. To depict spatial autocorrelation, landscape heterogeneity, spatial variability and patterns, Moran’I, experimental variograms and their variogram models were first analyzed and fitted to seven images of the studied area (Figure 6 and Table 3) [20]. Figure 6(a) shows correlograms of the Moran’s I (distance as the upper distance of a lag) in the seven events. All show positive spatial autocorrelation in short distance and negative ones in large distance, which is called gradient spatial pattern. Typhoons and earthquakes will disturb the spatial autocorrelation of the NDVI. Spatial correlations for all events among watershed land patches at distances on less than 2700 m are considerably positive. In the figure, the spatial autocorrelation of NDVI images in the area are highest on 1999/03/06 and are the lowest on 1999/10/31. Between the two dates, the Chi-Chi earthquake happened. Hence, the earthquake will cause the most serious disturbance and reduce the spatial autocorrelation. In Figure 6(b), the variogram models of the seven NDVI images for the study area are exponential models. The Sill (*C*_0_ + *C*) of NDVI images from high to low in the area are on 1999/10/31, 2000/11/27, 2004/11/19, 2003/12/17, 2001/11/20, 1999/03/06 and 1996/11/08. The sill is the upper limit that a variogram approaches at a large distance, and is a measure of the variability of the investigated variable: a higher sill corresponds to greater variability in the variable. The spatial variations of NDVI images increase considerably from 1996/11/08 to 1999/10/31 (after the Chi-Chi earthquake) in the area. The Nugget effects of NDVI images on 1999/10/31 are larger than those in other images. The nugget effect is exhibited by the apparent non-zero value of the variogram at the origin, which may be due to the small-scale variability of the investigated process and measured errors. Exponential models with large sills and large nugget effects NDVI images are indicative of significant spatial heterogeneous landscapes induced by the Chi-Chi earthquake in the area. Moreover, typhoons Xangsane and Dujuan also generated heterogeneous landscapes in the area [20]. Spatial correlations of NDVI over the watershed at distances less than 2,800 m are considerably positive. Spatial autocorrelation (Moran’s I) was used to delineate spatial variations in the landscape and class levels of the sub-watersheds before and after disturbances [25]. In the paper, the spatial correlation tendencies are similar but the changes before and after disturbances are significantly different. Moreover, variography results confirm that the impacts of disturbances on the watershed landscape pattern were cumulative, but were not always evident in space and time in the entire landscape [9,71]. Variography results illustrate that NDVI discontinuities between fields create a mosaic spatial structure resulting primarily from large disturbances, such as the Chi-Chi earthquake, in the study area. In addition, the landscape metrics examine the patterns of landscape fragmentation after the large disturbances on the watershed landscape. The landscape metrics also indicate that the disturbances and disturbance regime are characterized by a variety of attributes, including size, frequency, intensity, severity, and shape [27].

In this study, spatial analysis and modeling results indicate that large disturbances, such as the Chi-Chi earthquake, created extremely complex heterogeneous patterns across the landscape. Thus, a disturbance may affect some areas and disturbance severity often varies considerably within an affected area on the landscape level [25,45]. However, the earthquake and the typhoons impacted the fragmentation, shape, isolation, and interspersion of the patches. These results verify that disturbances create complex heterogeneous patterns across the landscape because the severity of the disturbances frequently varies considerably within the affected area. Disturbances are not the only destructive and restorative causes of modification of the geomorphic landscape or the structure and composition of the forest that occur between disturbances [72]. The spatial analysis results of NDVI images are sufficient to present landscape changes induced by disturbances, particularly via spatial structure, variability and spatial correlation. Previous studies [25,41,70] indicated that landslides in the Chenyulan watershed were impacted by the Chi-Chi earthquake; however, the effect of the earthquake decreased as the time after the Chi-Chi earthquake [70]. Landslides induced by earthquakes and typhoons have distinct spatial patterns [41]. Bare land and grassland have high potential to become landslides during the large disturbances [48]. The high-magnitude Chi-Chi earthquake created these landscape variations in space in the Chenyulan watershed [25].Moreover, typhoons significantly influence NDVI variations via the flow of accumulated rainfall and wind gradients [19]. Thus, the modeling results also prove the previous studies indicating that earthquake and subsequent rainstorms may cause divergent destruction of vegetation, and then this destruction may be influenced by the precipitation distribution and typhoon path.

Furthermore, the results from mean NDVI revealed vegetation recovery tendency and a stable plant growth cycle for the study area. The preview study shows the same results that the vegetation recovery rate reached more than half of (58.93%) original vegetation regeneration in the landslide areas over two years of monitoring and assessing [69]. The poor recovery locations were distributed mainly in mountain ridge, scoured slope base and acidic sulfate soil areas. The areas had lower recovery rates affected by the impacts of the slope and the property of the soil [69,73].

### 3.2. Simulation with Selected Samples for Multiple Images

cLHS is a stratified random procedure that provides an efficient way of sampling variables from their multivariate distributions. It provides a full coverage of range each variable by ensuring a good spread of the sampling points [12]. In the previous study [20], experimental variograms of cLHS samples with their NDVI values were constructed using the same lag interval to compare the spatial structures of the actual NDVI images. Lin *et al*. [20] listed the statistics and variogram for different samples from multiple NDVI images with 62,500 grids using the cLHS approach. The statistics for these that select more than 3,000 samples indicate the statistics obtained by cLHS can capture statistics of all actual NDVI images. The statistical and variogram analyses of cLHS samples also illustrate that the cLHS approach can be applied to select samples and capture the spatial structures of multiple historically accurate NDVI images [20]. The distributions of selected samples confirm that samples selected using cLHS provide a good coverage of the study area and are well spread and partially clustered in the study area [12]. These samples can be used in further monitoring and simulation to determine the impacts of disturbances on study landscapes in the latter.

**Figure 7.**
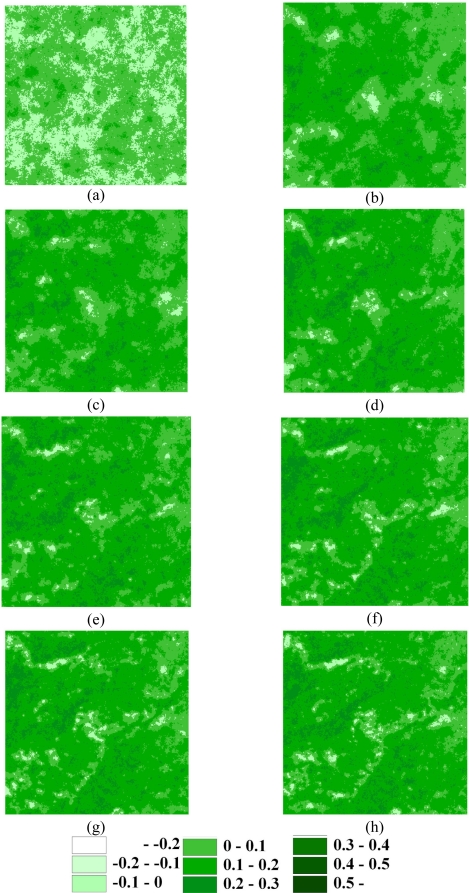
SGS simulated NDVI images based on (a) 100, (b) 500, (c) 1,000, (d) 2,000, (e) 3,000, (f) 5,000, (g) 7,000, (h) 10,000 on 1999/10/31.

**Figure 8.**
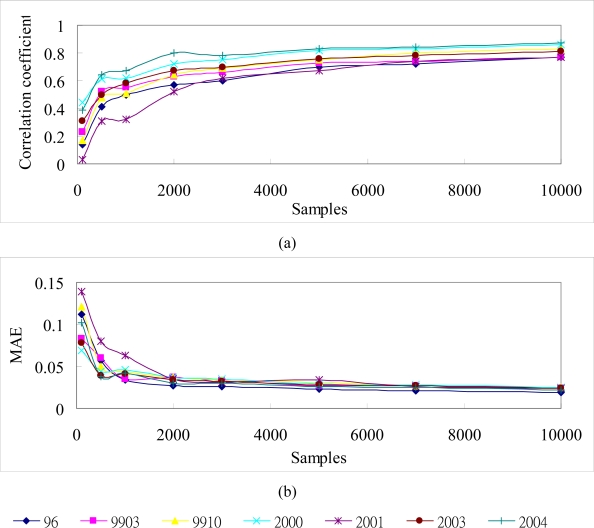
(a) Correlation coefficients and (b) MAE between the original data and SGS simulation data with different sampling data in seven events.

In this study, SGS simulation was performed based on the different samples for seven NDVI images in the area. Table 4 shows the statistics of the SGS simulations using 100, 500, 1,000, 2,000, 3,000, 5,000, 7,000, and 10,000 cLHS selected samples in the seven events. The comparison reveals that the statistics of these SGS simulations are matched to the original data. Over 3,000 samples, the statistics of these simulations are close to that of the original data. Figure 7 shows the average of 1,000 NDVI realizations produced by SGS simulations with 100, 500, 1,000, 2,000, 3,000, 5,000, 7,000, and 10,000 cLHS selected samples on 1999/10/31 for the area. It is proved that simulation based on more samples can increase the accuracy of SGS simulation, especially under the situation of more than 3,000 samples. The simulation with the efficient samples can map the spatial patterns of landscape after Chi-Chi earthquake. Figure 8(a) shows the correlation coefficients between the original data and simulation data of different sample numbers in seven events. The coefficients of sampling simulation maps are close to these of the original data when samples increase. After 5,000 samples, the correlation increases slowly. The results indicate that samples number increases a lot in applications, but the correlation increasing rate inclines to steady. Figure 8(b) shows the mean absolute error (MAE) between the referenced and simulated data. Results are consistent with that indicates the more the samples, the higher the match accuracy. SGS simulation based on sufficient samples can capture the spatial characteristics of landscape changes including spatial heterogeneity and variability.

**Figure 9.**
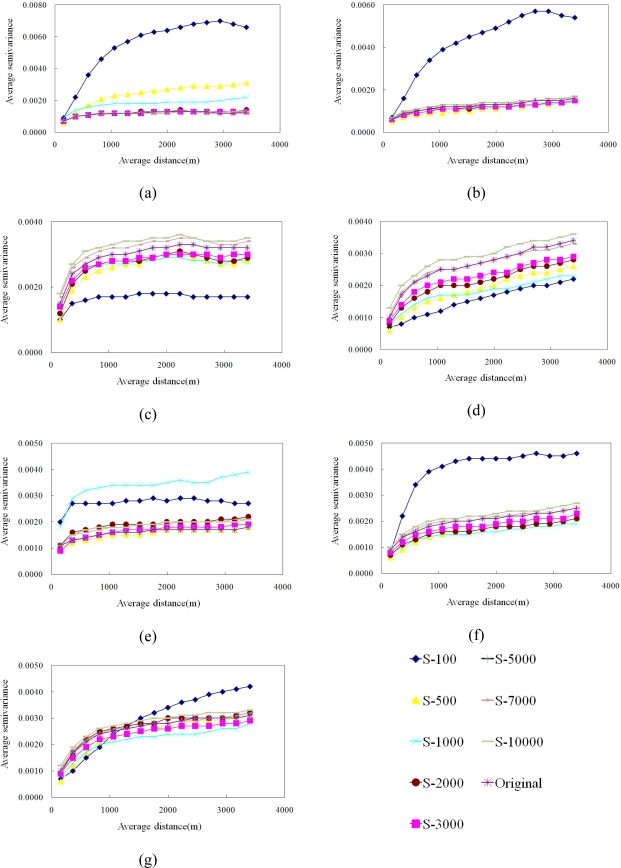
Experimental variograms of NDVI simulations using different samples on (a) 1996/11/08, (b) 1999/03/06, (c) 1999/10/31, (d) 2000/11/27, (e) 2001/11/20, (f) 2003/12/17, and (g) 2004/11/19.

**Figure 10.**
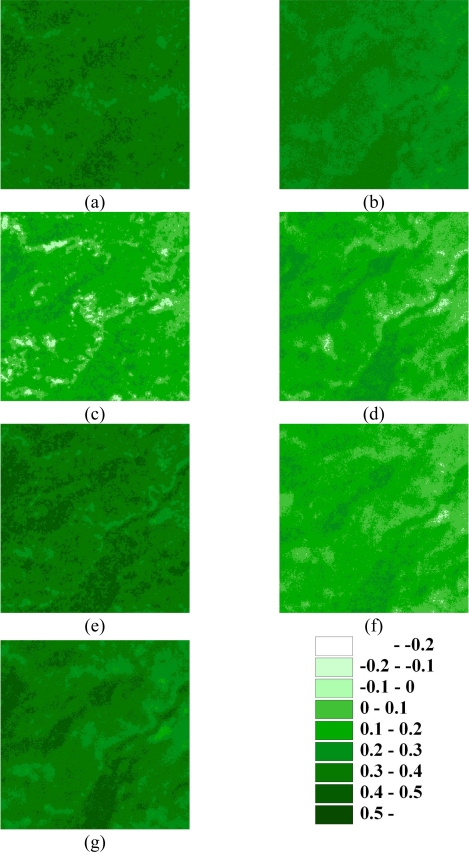
SGS simulated NDVI images based on 5,000 samples for area on (a) 1996/11/08, (b) 1999/03/06, (c) 1999/10/31, (d) 2000/11/27, (e) 2001/11/20, (f) 2003/12/17, and (g) 2004/11/19.

This study also presents the spatial analysis such as the variograms and landscape metrics to explore the relationship in different samplings. Figure 9 shows experimental variograms for 100, 500, 1,000, 2,000, 3,000, 5,000, 7,000 and 10,000 cLHS selected samples and original data on 1996/11/08, 1999/03/06, 1999/10/31, 2000/11/27, 2001/11/20, 2003/12/17 and 2004/11/19, respectively. These experimental variograms show that as the number of samples increased from 100 to 10,000, the ability of experimental variograms to capture the spatial structure of actual NDVI images increased. These variography results show that the cLHS and SGS approaches can simultaneously select samples from multiple NDVI images and simulate all NDVI spatial structures. Results show the variograms are good indicators of pattern identification of the NDVI spatial structures in this study. The variation in variogram patterns observed among watersheds show that the underlying spatial structure and landscape change induced by the disturbances. The variogram using less than 1,000 samples are poor spatial representation. From the statistical and spatial analysis, effective samples (5000 samples) can be selected by cLHS (only 8% of total) and replicate the statistical distribution and spatial structures of the NDVI from the original data. In final, Figure 10 shows NDVI maps produced by SGS simulations with 5,000 cLHS samples in the seven events of the area. The SGS results verify that the limits of spatial analysis and interpolations of landscape variables are based on semivariograms (or autocorrelation functions) solely, stressing the need to account for spatial patterns in highly heterogeneous landscapes after large physical disturbances [74]. Therefore, procedures for interpolation of NDVI must include information on spatial patterns, either directly from remotely sensed images or indirectly by sampling with sufficient spatial variables in the field that depend on the interest variable (assuming the sampling is too expensive in the field) [20,74]. The simulated NDVI images show that SGS and the cLHS approaches provide effective tools for monitoring, sampling and mapping landscape changes.

To illustrate how to deliver to field surveyors to website, this study set up a prototype based on OGC (Open Geospatial Consortium) to display the sampling data distribution. The OGC developed a series of standards for geospatial and location based services such as GML (Geography Markup Language), WMS (Web Map Service) and WFS (Web Feature Service).

**Figure 11.**
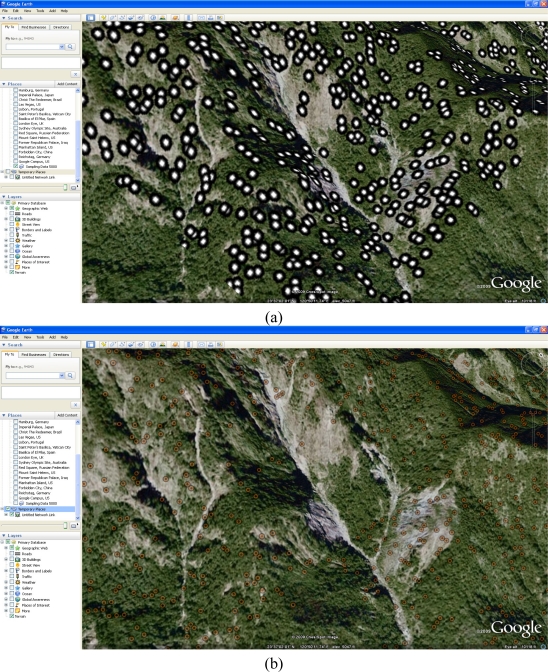
(a) Sampling data is overlaid on the latest image of Google Earth; (b) The server delivered a KML according to end-user's request on Google Earth.

The GML is a geospatial data standard that is neutral to commercial GIS software data format, as well as there is more and more GIS software complaining with GML. The WMS and WFS enable data providers to publish their by the approach that is based on IT standard and easy to implement. Figure 11(a) showed the sampling data is overlaid on the latest image of Google Earth. When end user sent a WMS request to the server, the server will response the image of sampling data. Google Earth is able to overlay the image from WMS response. Thus, end-users can browse the sampling data via Google Earth. Moreover, the server can response KML data that is a subset of GML. Figure 11(b) illustrated the server delivered a KML according to end-user's request and the KML is overlaid on Google Earth.

## 4. Conclusions

In the paper, multiple NDVI images, which can be generated almost immediately after the remotely sensed data are acquired, were used as the detection of landscape changes induced by a large disturbance. This study presents an effective framework that integrates cLHS, SGS, and spatial analysis in remotely sensed images for efficient monitoring, sampling, and mapping of the impacts from chronologically large disturbances on spatial characteristics of landscape changes including spatial structure, variability and heterogeneity.

The cLHS approach is an effective sampling approach for multiple NDVI images from the multivariate distributions to replicate the statistical distribution and spatial structures of the NDVI images. Using the spatial analysis such as Moran’I, and variography, SGS with sufficient samples generates multiple realizations and a realization average of NDVI, as well as captures the spatial variability and heterogeneity of disturbed landscapes. Spatial analysis of pre- and post-NDVI images of a large disturbance is essential for characterizing and quantifying the spatial variability, structure, and heterogeneity of landscapes induced by a disturbance. Moreover, landscape metrics have been proved effective in land-use change detection because they can characterize the differences between the events. The results illustrated that the impacts of large-physical disturbances on spatial variability existed and depended on disturbance magnitudes and paths, but were not always evident in spatiotemporal variability of landscapes in the study area.

In sum, the sufficient number of NDVI samples using cLHS (only 8% of total) can be applied to monitor the land cover change, which was induced by large physical disturbances. Then, the sampling data are demonstrated on the latest image of Google Earth by using Open Geospatial techniques. From the spatial analysis, the spatial simulations based on the sufficient samples are found to capture the spatial patterns of original NDVI distributions. Land cover changes in a watershed can alter hydrological processes and cause severe damages. When the typhoons came in the area, they brought about landslides and debris flows. Thus, the detection and concern about land cover change have benefit to soil and water conservation. This study will further research on the relationship between land cover change and hydrological processes.

## Figures and Tables

**Table 1.** Landscape metrics.

**Name**	**Equation**	**Note**
Number of patches (NP)	NP=*n_i_*	Patch size metrics
Mean patch size (MPS)	$\text{MPS}=\frac{1}{n_{i}}\sum_{j=1}^{n_{i}}a_{\mathrm{ij}}$	Patch size metrics
Patch Size Standard Deviation (PSSD)	$\mathrm{PSSD}=\sqrt{\frac{{\sum_{j=1}^{n}\left[a_{\mathrm{ij}}-\left(\frac{\sum_{j=1}^{n}a_{\mathrm{ij}}}{n_{j}}\right)\right]}^{2}}{n_{j}}}\left(\frac{1}{100000}\right)$	Patch size variability
Patch Size Coefficient of Variance (*PS*cov*)*	$\mathrm{PS}\text{cov}=\frac{\text{PSSD}}{\text{MPS}}(100)$	Patch size variability
Edge Density (ED)	$\text{ED}=\frac{\sum_{j=1}^{n}e_{\mathrm{ij}}}{A}(10000)$	Edge metrics
Mean shape index (MSI)	$\text{MSI}=\frac{\sum_{j=1}^{n_{i}}\frac{0.25p_{\mathrm{ij}}}{\sqrt{a_{\mathrm{ij}}}}}{n_{i}}$	Shape metrics
Mean nearest neighbor (MNN)	$\text{MNN}=\frac{\sum_{j=1}^{n_{i}}h_{\mathrm{ij}}}{n_{i}}$	Diversity metrics

where *n_i_* is the number of patches in land-use class *i; a_ij_* is the *j*^th^ patch area (ha.) inland-use class *i; m* is the total number of patch classes; *e_ik_* is the total length (m) of the edge between patch classes *i* and *k; p_ij_* is the *j*^th^ patch perimeter (m) in land-use class *i; h_ij_* is the distance (m) from the *j*^th^ patch to the nearest neighboring patch of the same class *i*, based on the edge-to-edge distance.

**Table 2.** Statistics of NDVI images.

**Date**	**Mean**	**Std.**	**Min.**	**Max.**	**Skewness**	**Kurtosis**
1996/11/08	0.36	0.04	0.11	0.48	−0.98	1.45
1999/03/06	0.32	0.04	0.13	0.43	−0.58	0.08
1999/10/31	0.14	0.07	−0.22	0.33	−1.23	1.35
2000/11/27	0.15	0.07	−0.14	0.35	−0.47	−0.30
2001/11/20	0.37	0.05	0.03	0.50	−1.03	1.34
2003/12/17	0.15	0.06	−0.12	0.33	−0.27	0.00
2004/11/19	0.35	0.06	0.05	0.54	−0.44	0.07

**Table 3.** Variogram models of NDVI images.

**Date**	**Model**	**Parameters**			**RSS**	***r^2^***
		**C0 (mg/kg)^2^**	**C0+C(mg/kg)^2^**	**R(m)**		
1996/11/08	Exp.	0.000138	0.001326	654	1.61E-08	0.953
1999/03/06	Exp.	0.000712	0.001814	4620	6.07E-08	0.945
1999/10/31	Exp.	0.000590	0.004440	564	1.68E-07	0.939
2000/11/27	Exp.	0.000186	0.004676	2646	2.47E-07	0.952
2001/11/20	Exp.	0.000121	0.002429	1281	5.62E-08	0.933
2003/12/17	Exp.	0.000126	0.003126	2298	1.57E-07	0.949
2004/11/19	Exp.	0.000116	0.003832	1680	1.19E-07	0.977

Exp.: Exponential model; Sph.: Spherical model; C0: Nugget; C0+C: Sill; R: Range; RSS: Residual Sums of Squares

**Table 4.** Statistics of SGS simulations with different samples from NDVI images.

**Samples**	**Date**	**Mean**	**Std**	**Min**	**Max**	**Skewness**	**Kurtosis**
100	1996/11/08	0.25	0.08	−0.03	0.44	−0.40	−0.42
	1999/03/06	0.24	0.07	0.00	0.41	−0.42	−0.27
	1999/10/31	0.02	0.04	−0.12	0.28	0.45	0.52
	2000/11/27	0.10	0.04	−0.06	0.29	−0.13	−0.23
	2001/11/20	0.02	0.05	−0.13	0.46	1.64	5.51
	2003/12/17	0.08	0.07	−0.12	0.29	0.17	−0.33
	2004/11/19	0.26	0.06	0.07	0.49	−0.08	−0.59
500	1996/11/08	0.36	0.05	0.11	0.52	−0.23	−0.07
	1999/03/06	0.30	0.04	0.15	0.42	−0.22	−0.09
	1999/10/31	0.12	0.05	−0.15	0.26	−0.42	−0.14
	2000/11/27	0.14	0.05	−0.05	0.30	−0.22	−0.41
	2001/11/20	0.29	0.04	0.09	0.46	−0.15	0.30
	2003/12/17	0.13	0.04	−0.05	0.31	−0.29	−0.11
	2004/11/19	0.36	0.05	0.11	0.52	−0.23	−0.07
1000	1996/11/08	0.35	0.05	0.10	0.49	−0.83	1.55
	1999/03/06	0.26	0.04	0.12	0.41	−0.04	0.09
	1999/10/31	0.13	0.05	−0.12	0.29	−0.49	0.09
	2000/11/27	0.13	0.05	−0.05	0.30	−0.13	−0.13
	2001/11/20	0.31	0.06	0.02	0.50	−0.64	0.61
	2003/12/17	0.12	0.04	−0.04	0.29	−0.14	−0.04
	2004/11/19	0.33	0.05	0.12	0.51	−0.21	−0.05
2000	1996/11/08	0.37	0.04	0.14	0.52	−0.56	0.77
	1999/03/06	0.29	0.04	0.14	0.42	−0.11	−0.11
	1999/10/31	0.14	0.05	−0.20	0.31	−0.70	0.43
	2000/11/27	0.14	0.05	−0.1	0.35	−0.10	−0.36
	2001/11/20	0.36	0.05	0.07	0.52	−0.64	1.07
	2003/12/17	0.13	0.04	−0.05	0.33	−0.10	−0.21
	2004/11/19	0.37	0.06	0.10	0.54	−0.22	−0.19
3000	1996/11/08	0.37	0.04	0.18	0.49	−0.57	0.65
	1999/03/06	0.29	0.04	0.15	0.42	−0.17	−0.08
	1999/10/31	0.14	0.05	−0.19	0.33	−0.76	0.58
	2000/11/27	0.14	0.05	−0.04	0.32	−0.18	−0.29
	2001/11/20	0.37	0.04	0.07	0.51	−0.59	0.64
	2003/12/17	0.14	0.05	−0.11	0.31	−0.18	−0.09
	2004/11/19	0.35	0.05	0.12	0.52	−0.15	−0.19
5000	1996/11/08	0.37	0.04	0.15	0.48	−0.58	0.60
	1999/03/06	0.30	0.04	0.14	0.43	−0.19	−0.09
	1999/10/31	0.14	0.06	−0.20	0.29	−0.81	0.61
	2000/11/27	0.15	0.06	−0.10	0.31	−0.29	−0.29
	2001/11/20	0.33	0.04	0.03	0.48	−0.20	0.16
	2003/12/17	0.14	0.05	−0.10	0.31	−0.22	−0.16
	2004/11/19	0.35	0.05	0.10	0.52	−0.26	−0.10
7000	1996/11/08	0.37	0.04	0.12	0.49	−0.67	0.81
	1999/03/06	0.30	0.04	0.13	0.42	−0.25	−0.05
	1999/10/31	0.14	0.06	−0.18	0.32	−0.84	0.55
	2000/11/27	0.14	0.06	−0.10	0.33	−0.26	−0.30
	2001/11/20	0.37	0.05	0.04	0.51	−0.70	0.75
	2003/12/17	0.14	0.05	−0.10	0.31	−0.24	−0.07
	2004/11/19	0.35	0.05	0.07	0.52	−0.27	−0.05
10000	1996/11/08	0.37	0.04	0.16	0.48	−0.69	0.77
	1999/03/06	0.30	0.04	0.14	0.43	−0.25	−0.14
	1999/10/31	0.14	0.06	−0.17	0.33	−0.91	0.77
	2000/11/27	0.15	0.06	−0.10	0.32	−0.27	−0.36
	2001/11/20	0.37	0.05	0.09	0.50	−0.75	0.82
	2003/12/17	0.14	0.05	−0.10	0.31	−0.22	−0.20
	2004/11/19	0.35	0.07	0.06	0.54	−0.27	−0.11

## References

[1] Cihlar J., Latifovic R., Chen J., Beaubien J., Li Z. (2000). Selecting representative high resolution sample images for land cover studies. Part 1: Methodology. Remote Sens. Environ.

[2] Turner M.G. (1987). Spatial simulation of landscape changes in Georgia: A comparison of 3 transition models. Landscape Ecol.

[3] Petit C., Scudder T., Lambin E. (2001). Quantifying processes of land-cover change by remote sensing: resettlement and rapid land-cover changes in south-eastern Zambia. Int. J. Remote Sens.

[4] Lanfredi M., Simoniello T., Macchiato M. (2004). Temporal persistence in vegetation cover changes observed from satellite: Development of an estimation procedure in the test site of the Mediterranean Italy. Remote Sens. Environ.

[5] Sellers P.J. (1997). Modeling the exchange of energy, water, and Carbon between continents and atmosphere. Science.

[6] Southworth J., Munroe D., Nagendra H. (2004). Land cover change and landscape fragmentation - comparing the utility of continuous and discrete analyses for a western Honduras region. Agr. Ecosyst. Environ.

[7] Teillet P.M., Staenz K., Williams D.J. (1997). Effects of spectral, spatial, and radiometric characteristics on remote sensing vegetation indices of forested regions. Remote Sens. Environ.

[8] Garrigues S., Allard D., Baret F. (2007). Using first- and second-order variograms for characterizing landscape spatial structures from remote sensing imagery. IEEE Geosci. Remote Sens. Soc.

[9] Sellers P.J. (1985). Canopy reflectance, photosynthesis and transpiration. Int. J. Remote Sens.

[10] Cakir H.I., Khorram S., Nelson S.A.C. (2006). Correspondence analysis for detecting land cover change. Remote Sens. Environ.

[11] Biging G., Colby D., Congalton R.G., Lunetta R.S., Eldvidge C.D. (1998). Remote sensing change detection: environmental monitoring methods and applications. Sampling Systems for Change Detection Accuracy Assessment.

[12] McKay M.D., Beckman R.J., Conover W.J. (1979). A comparison of three methods for selecting values of input variables in the analysis of output from a computer code. Technometrics.

[13] Iman R.L., Conover W.J. (1980). Small sample sensitivity analysis techniques for computer models, with an application to risk assessment. Commun. Statist. Theory Meth.

[14] Minasny B., McBratney A.B. (2006). A conditioned Latin hypercube method for sampling in the presence of ancillary information. Comput. Geosci.

[15] Xu C., He H.S., Hu Y., Chang Y., Li X., Bu R. (2005). Latin hypercube sampling and geostatistical modeling of spatial uncertainty in a spatially explicit forest landscape model simulation. Ecol. Model.

[16] Lin Y.P., Chang T.K., Teng T.P. (2001). Characterization of soil lead by comparing sequential Gaussian simulation, simulated annealing simulation and kriging methods. Environ. Geol.

[17] Zhao Y.C., Shi X.Z., Yu D.S., Wang H.J., Sun W.X. (2005). Uncertainty assessment of spatial patterns of soil organic carbon density using sequential indicator simulation, a case study of Hebei province, China. Chemosphere.

[18] Zhao Y.C., Xu X.H., Huang B., Sun W.X., Shao X.X., Shi X.Z., Ruan X.L. (2007). Using robust kriging and sequential Gaussian simulation to delineate the copper- and lead-contaminated areas of a rapidly industrialized city in Yangtze River Delta, China. Environ. Geol.

[19] Lin Y.P., Yen M.H., Deng D.P., Wang Y.C. (2008). Geostatistical approaches and optimal additional sampling schemes for spatial patterns and future samplings of bird diversity. Global Ecol. Biogeogr.

[20] Lin Y.P., Chu H.J., Wang C.L., Yu H.H., Wang Y.C. (2009). Remote sensing data with the conditional latin hypercube sampling and geostatistical approach to delineate landscape changes induced by large chronological physical disturbances. Sensors.

[21] Deutsch C.V., Journel A.G. (1992). GSLIB. Geostatistical Software Library and User’s Guide.

[22] Minasny B., McBratney A.B., Walvoort D.J.J. (2007). The variance quadtree algorithm: Use for spatial sampling design. Comp. Geosci.

[23] Garrigues S., Allard D., Baret F., Weiss M. (2006). Quantifying spatial heterogeneity at the landscape scale using variograrn models. Remote Sens. Environ.

[24] Curran P.J., Atkinson P.M. (1998). Geostatistics and remote sensing. Prog. Phys. Geog.

[25] Lin Y.P., Chang T.K., Wu C.F., Chiang T.C., Lin S.H. (2006). Assessing impacts of typhoons and the ChiChi earthquake on Chenyuland watershed landscape patterns in central Taiwan using landscape metrics. Environ. Manage.

[26] Garrigues S., Allard D., Baret F. (2008). Modeling temporal changes in surface spatial heterogeneity over an agricultural site. Remote Sens. Environ.

[27] Turner M.G., Gardner R.H., O’Neill R.V. (2001). Landscape Ecology in Theory and Practice: Pattern and Process.

[28] Walsh S.J., Crawford T.W., Welsh W.F., Crews-Meyer K.A. (2001). A multiscale analysis of LULC and NDVI variation in Nang Rong district, northeast Thailand. Agr. Ecosyst. Environ.

[29] Stefanov W.L., Netzband M. (2005). Assessment of ASTER land cover and MODIS NDVI data at multiple scales for ecological characterization of an arid urban center. Remote Sens. Environ.

[30] Huang S.L., Wang S.H., Budd W.W. (2009). Sprawl in Taipei’s peri-urban zone: Responses to spatial planning and implications for adapting global environmental change. Landscape Urban Plan.

[31] McGarigal K., Marks B.J. (1995). FRAGSTATS: Spatial Pattern Analysis Program for Quantifying Landscape Structure.

[32] Leitão A.B., Ahern J. (2002). Applying landscape ecological concepts and metrics in sustainable landscape planning. Landscape Urban Plan.

[33] Corry R.C., Nassauer J.I. (2005). Limitations of using landscape pattern indices to evaluate the ecological consequences of alternative plans and designs. Landscape Urban Plan.

[34] Keefer D.K. (1984). Landslides caused by earthquakes. Geol. Soc. Am. Bul.

[35] Lin Y.B., Lin Y.P, Deng D.P. (2008). Integrating remote sensing data with directional two-dimension wavelet analysis and open geospatial techniques for effective disaster monitoring and management. Sensors.

[36] Keefer D.K. (1994). The importance of earthquake-induced landslides to long term slope erosion and slope-failure hazards in seismically active regions. Geomorphology.

[37] Chen S.C., Wu C.H. (2006). Slope stabilization and landslide size on Mt. 99 Peaks after Chichi Earthquake in Taiwan. Environ. Geol.

[38] Lee M.F., Lin T.C., Vadeboncoeur M.A., Hwong J.L. (2008). Remote sensing asseeement of forest damage in relation to the 1996 strong typhoon Herb at Lienhuachi Experimental Forest, Taiwan. Forest Ecol. Manag.

[39] Tsutsui K., Rokugawa S., Nakagawa H., Tsutsui K., Miyazaki S., Cheng C.-T., Shiraishi T., Yang S.D. (2007). Detection and volume estimation of large-scale landslides based on elevation-change analysis using dems extracted from high-resolution satellite stereo imagery. IEEE Trans. Geosci. Remote Sens.

[40] Chen C.Y., Lin L.Y., Yu F.C., Lee C.S., Tseng C.C., Wang A.H., Cheung K.W. (2007). Improving debris flow monitoring in Taiwan by using high-resolution rainfall products from QPESUMS. Nat. Hazards.

[41] Lin G.W., Chen H., Chen Y.H., Horng M.J. (2008). Influence of typhoons and earthquakes on rainfall-induced landslides and suspended sediments discharge. Eng. Geol.

[42] Lin C.W., Shieh C.L., Yuan B.D., Shieh Y.C., Liu S.H., Lee S.Y. (2003). Impact of Chi-Chi earthquake on the occurrence of landslides and debris flows: example from the Chenyulan River watershed, Nantou, Taiwan. Eng. Geol.

[43] Foster D.R., Knight D.H., Franklin J.F. (1998). Landscape patterns and legacies resulting from large, infrequent forest disturbances. Ecosystems.

[44] Swanson F.J., Johnson S.L., Gregory S.V., Acker S.A. (1998). Flood disturbance in a forested mountain landscape. Bioscience.

[45] Turner M.G., Dale V.H. (1998). Comparing large, infrequent disturbances: what have we learned?. Ecosystems.

[46] Millward A.A., Kraft C.E. (2004). Physical influences of landscape on a large-extent ecological disturbance: the northeastern North American ice storm of 1998. Landscape Ecol.

[47] Lin C.W., Liu S.H., Lee S.Y., Liu C.C. (2006). Impacts of the Chi-Chi earthquake on subsequent rainfall-induced landslides in central Taiwan. Eng. Geol.

[48] Hong N.M., Chu H.J., Lin Y.P., Deng D.P. (2009). Effects of land cover changes induced by large physical disturbances on hydrological responses in Central Taiwan. Environ. Monit. Assess.

[49] Roger B., Yu T.T. (2000). The morphology of thrust faulting in the 21 September 1999, Chi-Chi, Taiwan earthquake. J. Asian Earth Sci.

[50] Central Weather Bureau (CWB) http://www.cwb.gov.tw/.

[51] Central Weather Bureau (2000). Report on Typhoons in 2000.

[52] Central Weather Bureau (2001). Report on Typhoons in 2001.

[53] Central Weather Bureau (CWB) (2008). Typhoon Database.

[54] Jensen T.R. (1996). Introductory Digital Image Processing: A Remote Sensing Perspective.

[55] Elkie P.C., Rempel R.S., Carr A.P. (1999). Patch Analyst User Manual: A Tool for Quantifying Landscape Structure.

[56] Fredericks A.K., Newman K.B. (1998). A comparison of the sequential Gaussian and Markov-Bayes simulation methods for small samples. Math. Geol.

[57] Lin C.Y., Wu J.P., Lin W.T. (2001). The priority of revegetation for the landslides caused by the catastrophic Chi-Chi earthquake at ninety-nine Peaks in Nantoun area. J. Chinese Soil Water Cons.

[58] Kyriakidis P.C., Hunsaker C.T., Goodchild M.F., Friedl M.A., Case T.J. (2001). Geostatistical models of uncertainty for spatial data, Spatial uncertainty in ecology: implications for remote sensing and GIS application.

[59] Legendre P. (1993). Spatial autocorrelation: trouble or new paradigm?. Ecology.

[60] Moran P.A.P. (1950). Notes on continuous stochastic phenomena. Biometrika.

[61] Legendre P., Legendre L. (1998). Numerical Ecology.

[62] Senay G.B., Elliott R.L. (2001). Combining AVHRR-NDVI and landuse data to describe temporal and spatial dynamics of vegetation. For Ecol Manage.

[63] Birky A.K. (2001). NDVI and a sample model of deciduous forest seasonal dynamics. Ecol. Model.

[64] Ward D., Phinn S.R., Murray A.T. (2000). Monitoring growth in rapidly urbanizing areas using remotely sensed data. Prof. Geog.

[65] Akiwumi F.A., Butler D.R. (2008). Mining and environmental change in Sierra Leone, West Africa: A remote sensing and hydrogeomorphological study. Environ. Monit. Assess.

[66] Giriraj A., Irfan-Ullah M., Murthy M.S.R., Beierkuhnlein C. (2008). Modelling spatial and temporal forest cover change patterns (1973–2020): A case study from South Western Ghats (India). Sensors.

[67] Fox D.M., Maselli F., Carrega P. (2008). Using SPOT images and field sampling to map burn severity and vegetation factors affecting post forest fire erosion risk. Catena.

[68] Zomeni M., Tzanopoulos J., Pantis J.D. (2008). Historical analysis of landscape change using remote sensing techniques: An explanatory tool for agricultural transformation in Greek rural areas. Landscape Urban Plan.

[69] Lin W.-T., Chou W.-C., Lin C.-Y., Huang P.-H., Tsai J.-S. (2005). Vegetation recovery monitoring and assessment at landslides caused by earthquake in Central Taiwan. For. Ecol. Manage.

[70] Chang K.T., Chiang S.H., Hsu M.L. (2007). Modeling typhoon- and earthquake- induced landslides in a mountains watershed using logistic regression. Geomorphology.

[71] Lin W.T., Lin C.Y., Chou W.C. (2006). Assessment of vegetation recovery and soil erosion at landslides caused by a catastrophic earthquake: a case study in Central Taiwan. Ecol. Eng.

[72] Scatena F.N., Lugo A.E. (1995). Geomorphology, disturbance, and the soil and vegetation of two subtropical wet steepland watersheds of Puerto Rico. Geomorphology.

[73] Lin C.Y., Lo H.M., Chou W.C., Lin W.T. (2004). Vegetation recovery assessment at the Jou-Jou Mountain landslide area caused by the 921 Earthquake in Central Taiwan. Ecol. Model.

[74] Lobo A., Moloney K., Chic O., Chiariello N. (1998). Analysis of fine-scale spatial pattern of a grassland from remotely-sensed imagery and field collected data. Landscape Ecol.

